# Severe *Saprochaete capitata* fungemia presenting as micafungin breakthrough hepatosplenic lesions in an immunocompromised patient: case report

**DOI:** 10.1128/asmcr.00071-25

**Published:** 2025-09-03

**Authors:** Hannakate Lichota, Eric Bhaimia, Graeme Forrest, Nicholas M. Moore

**Affiliations:** 1 Rush University Medical Center, Chicago, Illinois, USA; Pattern Bioscience, Austin, Texas, USA

**Keywords:** *Geotrichum capitatum*, *Magnusiomyces capitatus*, *Blastoschizomyces capitatus*, hepatosplenic fungal infection, echinocandin resistance, geotrichum, immunosuppressed host, invasive fungal infections, IFI, breakthrough fungal infection

## Abstract

**Background:**

*Saprochaete capitata* (*S. capitata*, also known as *Magnusiomyces capitatus*), a yeast-like fungus commonly isolated from soil, is an emerging infection capable of disseminated infection in immunocompromised hosts, including those with hematologic malignancy.

**Case Summary:**

A 70-year-old female admitted for induction therapy for myelodysplastic syndrome (MDS) developed persistent neutropenic fever while on antibacterial and micafungin prophylaxis. Blood cultures revealed high-grade *S. capitata* fungemia with imaging findings of hepatosplenic involvement in the setting of a central venous catheter and interatrial shunt. The patient was successfully treated with a combination of voriconazole, amphotericin B 3 mg/kg daily, and flucytosine. She was transitioned to voriconazole monotherapy after blood culture clearance and resolution of fever.

**Conclusion:**

*S. capitata* may be associated with severe, disseminated infection in patients with hematologic malignancy. While there is a lack of evidence to guide optimal treatment, the organism appears to exhibit resistance to echinocandins. Expert opinion favors voriconazole as empiric treatment with the addition of intravenous liposomal amphotericin B for severe infections.

## INTRODUCTION

Invasive fungal infections (IFI) represent a serious complication of prolonged neutropenia following treatment in patients with hematologic malignancies. Despite the use of routine triazole prophylaxis, rare fungal pathogens may cause breakthrough infection of the bloodstream and organs, with high morbidity and mortality (1). Management of fungal infection due to non-candidal species, such as *Saprochaete*, *Trichosporon*, or *Pichia*, is further complicated by a lack of antimicrobial susceptibility breakpoints to guide effective antifungal therapy. *Saprochaete capitata* (formerly known as *Magnusiomyces capitatus*, *Geotrichum capitatum*, or *Blastoschizomyces capitatus*) is one such pathogen. This rare yeast has been associated with invasive fungal infections in immunocompromised populations. We discuss the case of a neutropenic host who developed *S. capitata* fungemia with seeding of deep organs and provide a discussion on pathogen characteristics and antifungal management.

*S. capitata* is a yeast found in soil and may colonize the skin and respiratory and gastrointestinal tracts through environmental exposure (2). Reservoirs include sand, soil, plant material, such as wood pulp, and dairy products (2, 3). *S. capitata* colonies mature quickly (typically within 5 days) on a variety of culture media, including Sabouraud dextrose agar (at 37°C) or corn meal-Tween 80 agar at 25°C where it exhibits variable morphology, including pseudohyphae, true hyphae, anneliconidia, and arthroconidia at temperatures up to 42°C (4). *S. capitata* is a urease-negative yeast that utilizes only glucose and galactose and is cycloheximide resistant (4). Macroscopically, typical colonies are white or cream, shiny or opaque, and often wrinkled (4). It can be difficult to distinguish *Saprochaete* from *Trichosporon* and *Geotrichum* species without using matrix-assisted laser desorption/ionization time-of-flight (MALDI-TOF) mass spectrometry or internal transcribed spacer (ITS) sequencing. Commercial mass spectrometry systems may identify this isolate as *G. capitatum* or *B. capitatus* depending on the system and database version utilized. Morphologic differences between similar fungi may provide helpful clues to identification. For example, *Trichosporon* is classically described as producing hyphae and pseudohyphae with blastoconidia singly or in short chains, and arthroconidia can form on older cultures. *Geotrichum* produces coarse hyphae but not pseudohyphae that segment into rectangular arthroconidia with rounded ends. Blastoconidia are absent, which helps to differentiate *Geotrichum* from *Trichosporon* (5) (Table 1) (6, 7).

**TABLE 1:** Differentiating between similar fungi : *S. capitata, Trichosporon*, and *Geotrichum* spp.^*a*^

Characteristic/feature	*Saprochaete capitata*	*Trichosporon* spp.	*Geotrichum* spp.
Microscopic morphology on corn meal-Tween agar at 25°C:			
Hyphae	+	+	+
Pseudohyphae	+	+	=
Blastoconidia	+	+	=
Arthroconidia	v	+	+
Annelloconidia	+	=	=
Urease	=	+	=
Assimilation of the following carbohydrates:			
Glucose	+	+	+
Maltose	=	+ (v)	=
Sucrose	=	+ (v)	=
Lactose	=	+	=
Galactose	+	+ (v)	+
Xylose	=	+	+

^*a*^ +, positive; =, negative; v, variable.

## CASE PRESENTATION

**Fig 1:**
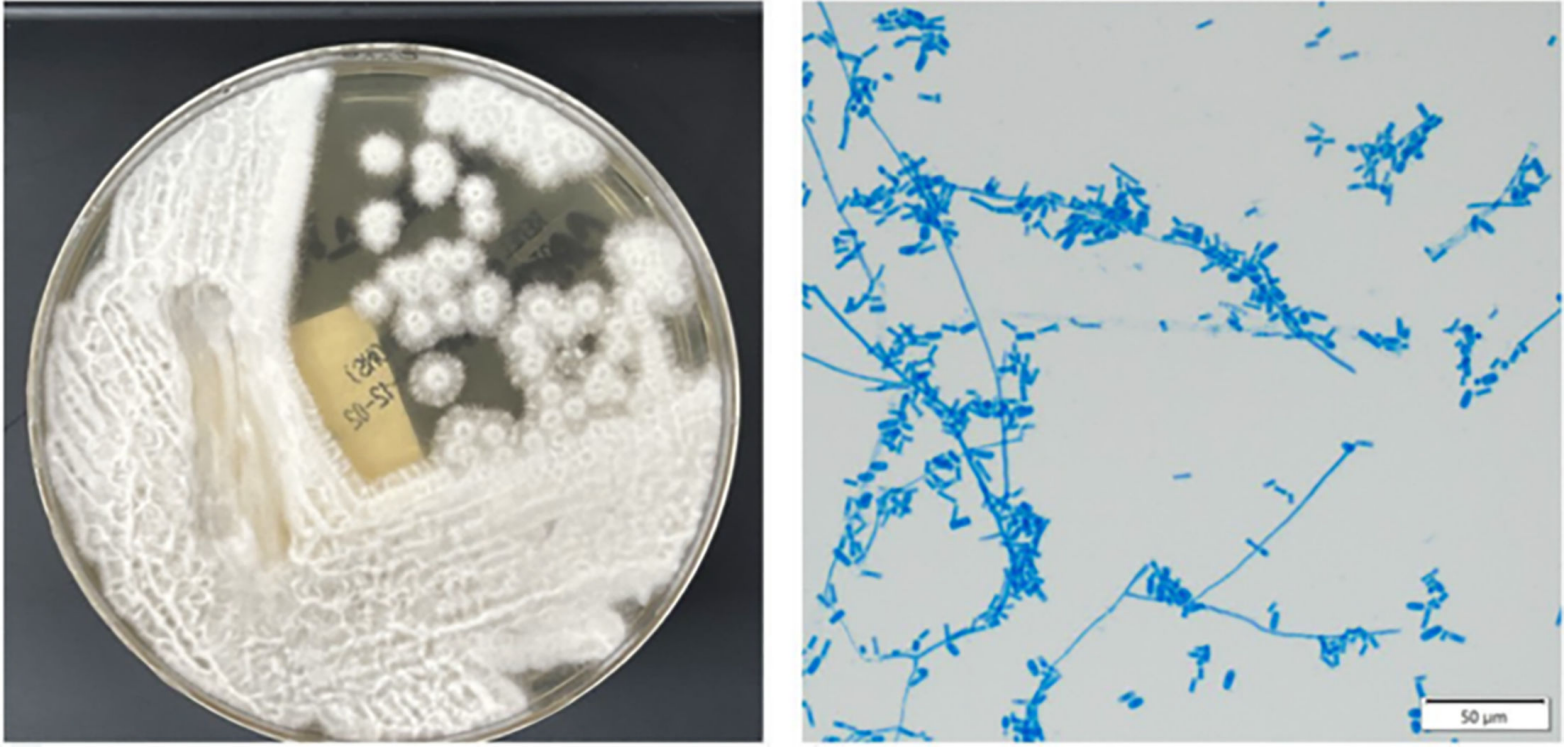
Growth of *S. capitata* on Sabouraud dextrose agar (left) after 5 days of incubation at 30°C in ambient air produced white, fuzzy colonies. The microscopic morphology stained with lactophenol cotton blue (right) highlights the appearance of oblong annelloconidia clustered at the tips of the annellides, which are located along or at the tips of the hyphae. This organism has been demonstrated to produce annelloconidia (6, 7).

A 70-year-old female with high-risk myelodysplastic syndrome status post-induction cytarabine/mitoxantrone via a right internal jugular catheter developed neutropenic fevers to 39.5°C on day 12 of admission despite prophylactic micafungin 50 mg IV daily. The patient’s social history was notable for gardening and recent home demo. Initial workup included blood cultures and computed tomography (CT) of the chest, abdomen, and pelvis, which were negative and unremarkable, respectively. Blood cultures were repeated daily for persistent fevers with growth of yeast at 48 h on hospital day 14. The organism grew on Sabouraud dextrose agar (Fig. 1) and was identified as *S. capitata* via MALDI-TOF mass spectrometry (Vitek MS, bioMérieux, Knowledge Base 3.2) on hospital day 15, prompting removal of the indwelling catheter, increase in micafungin dose to 100 mg daily, and addition of oral voriconazole 6 mg/kg load, followed by 4 mg/kg twice daily.

**Fig 2:**
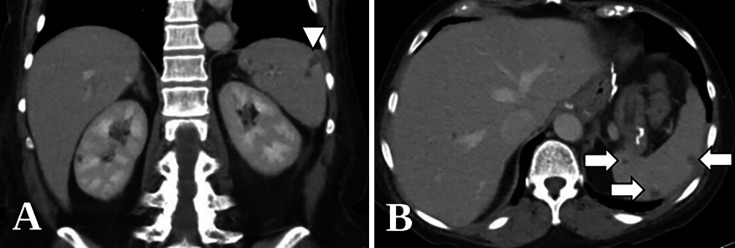
Coronal (**A**) and axial (**B**) computed tomographic imaging with contrast of the abdomen demonstrating wedge-shaped infarct (arrowhead) and splenic hypodensities (arrows), respectively.

The patient remained febrile to 41°C with continued isolation of *S. capitata* in all blood cultures from hospital days 12–15, at which time intravenous liposomal amphotericin B (LAmB) 3 mg/kg daily plus flucytosine (5-FC) 200 mg/kg q8h was started, and micafungin was discontinued. On hospital day 17, repeat CT imaging of the chest, abdomen, and pelvis revealed new subcentimeter hypodense lesions throughout the liver with similar hypodensities and a wedge-shaped infarct in the spleen (Fig. 2). Transthoracic echocardiogram (TTE) revealed an interatrial shunt without evidence of valvular vegetations to raise the concern for endocarditis. The patient’s blood cultures cleared after 2 days of LAmB and 5-FC, and she experienced neutrophil recovery with absolute neutrophil count (ANC) greater than 500 cells/µL 6 days later. In total, she received 1 week of combination LAmB and 5-FC.

**Fig 3:**
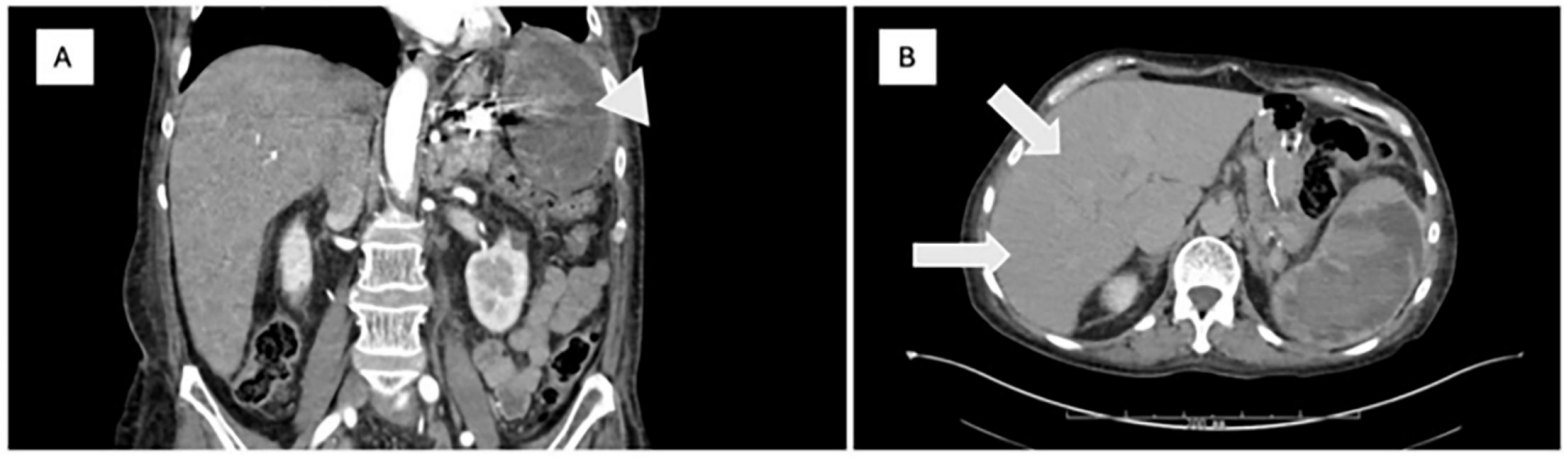
Coronal (**A**) and axial (**B**) computed tomographic imaging with contrast of the abdomen demonstrating splenic necrosis (arrowhead) and hepatic hypodensities (arrows), respectively.

Ultimately, the patient was transitioned to voriconazole 150 mg BID based on serum level of 1.6 ug/mL, with duration pending clinical response to therapy as well as decisions surrounding further treatment for MDS. Antifungal susceptibility revealed minimum inhibitory concentrations (MIC) of 1, 4, 1, and 0.125 µg/mL for amphotericin B, caspofungin, micafungin, and voriconazole, respectively. The patient was discharged to an acute rehabilitation facility on hospital day 35. She was readmitted 3 days later with fever and anemia with CT angiography findings of splenic artery mycotic aneurysm requiring embolization with resultant splenic necrosis. Repeat CT of the abdomen (Fig. 3) at this time also demonstrated innumerable hepatic hypodensities felt to represent a sequela of prior high-grade fungemia in the setting of immune reconstitution. Repeat blood cultures were without growth, and the patient was again discharged on voriconazole, with duration pending treatment response. Upon outpatient follow-up, she endorsed visual floaters on voriconazole, prompting transition to isavuconazole 372 mg daily. AML treatment consisting of decitabine/cedazuridine and venetoclax was further complicated by recurrent ESBL *Escherichia coli* bacteremia of presumed gut source and *Alternaria* invasive fungal rhinosinusitis requiring surgical debridement. She ultimately opted for a transition to hospice care and passed 7 months following her diagnosis of *S. capitata* infection.

## DISCUSSION

Patients with prolonged neutropenia, as seen in hematologic malignancies, are at risk of infection due to *S. capitata* (8–10). In some studies, risk of mortality due to invasive *Saprochaete* infection has approximated 65% with a predilection for organ involvement (3, 10, 11). While antifungal prophylaxis may reduce the incidence of an IFI, breakthrough infection with *Saprochaete* in patients who received echinocandin prophylaxis portends poor outcomes (8, 10–12). In addition to *Saprochaete*, other yeasts and yeast-like fungi demonstrating echinocandin breakthrough infection include *Cryptococcus*, *Trichosporon*, *Rhodutorula*, *Malassezia*, *Saccharomyces*, *Nakaseomyces* (formerly *Candida*) *glabrata*, and *Pichia kudriavzevii* (formerly *Candida krusei*) (13). It is speculated that *Saprochaete* exhibits echinocandin resistance given elevated MIC values; however, there are insufficient data to clarify if this represents intrinsic or acquired resistance (6, 14). Further complicating clinical management is the lack of antimicrobial susceptibility testing (AST) interpretive criteria; MIC breakpoints have not yet been established for *Saprochaete*, and there are no Food and Drug Administration-cleared or -approved commercial methods available for AST (1, 11). *In vitro* susceptibility data of *S. capitata* isolates from blood cultures of fungemic patients have described patterns of elevated MICs for echinocandins (MIC > 2 µg/mL) compared to lower breakpoints for triazoles (MIC below 1 µg/mL) in small case series, which were further substantiated in a larger case series of 34 patients (6, 11). *In vitro* antifungal activity reveals lower MIC values for voriconazole, followed by posaconazole and itraconazole (6). Contrasting this is an elevated MIC for echinocandins, particularly micafungin (6). While difficult to interpret given limitations of available breakpoints, the elevated MIC values of *Saprochaete* isolates are suspected to portend resistance. In the case of our patient, echinocandin resistance was suspected, leading to the use of three antifungals and eventual voriconazole monotherapy after clinical improvement and review of the available literature. Based on expert opinion, the European Society of Clinical Microbiology and Infectious Diseases (ESCMID) 2014 guidelines recommend LAmB with or without 5-FC as empiric therapy (9). This case illustrates the potential for severe *S. capitata* infection introduced via a central venous catheter with hepatosplenic involvement, presenting as an echinocandin breakthrough IFI in a patient experiencing prolonged neutropenia.
